# Diet, Microbiome, and Cancer Immunotherapy—A Comprehensive Review

**DOI:** 10.3390/nu13072217

**Published:** 2021-06-28

**Authors:** Michał Szczyrek, Paulina Bitkowska, Patryk Chunowski, Paulina Czuchryta, Paweł Krawczyk, Janusz Milanowski

**Affiliations:** Department of Pneumology, Oncology and Allergology, Medical University of Lublin, 20-090 Lublin, Poland; paulinabitkowska1@gmail.com (P.B.); patrykchunowski@gmail.com (P.C.); paulinaczuchryta651@gmail.com (P.C.); krapa@poczta.onet.pl (P.K.); janusz.milanowski@umlub.pl (J.M.)

**Keywords:** human intestinal microbiota, faecal microbiota transplant, immunotherapy, immune checkpoint blockade, nutrition, diet

## Abstract

The immune system plays a key role in cancer suppression. Immunotherapy is widely used as a treatment method in patients with various types of cancer. Immune checkpoint blockade using antibodies, such as anti-PD-1, anti-PD-L1, and anti-CTLA-4, is currently gaining popularity. A systematic literature search was executed, and all available data was summarized. This review shows that specific dietary patterns (such as, e.g., animal-based, vegetarian, or Mediterranean diet) alter the gut microbiome’s composition. An appropriate intestinal microbiota structure might modulate the function of human immune system, which affects the bodily anti-cancer response. This paper shows also that specific bacteria species inhabiting the gastrointestinal tract can have a beneficial influence on the efficacy of immunotherapy. Antibiotics weaken gut bacteria and worsen the immune checkpoint blockers’ efficacy, whereas a faecal microbiota transplant or probiotics supplementation may help restore bacterial balance in the intestine. Other factors (like vitamins, glucose, or BMI) change the cancer treatment response, as well. This review demonstrates that there is a strong association between one’s diet, gut microbiome composition, and the outcome of immunotherapy. However, further investigation on this subject is required.

## 1. Introduction

According to the current knowledge, the immune system plays an extremely important role in the pathogenesis of cancer [1]. The immune system can prevent tumor formation through the elimination of oncogenic viruses and inflammation-causing pathogens. It can also fight cancer development through a tumor immune surveillance, which is based on recognizing precancerous or cancerous cells and removing them before they cause any damage [2]. According to Sir Frank Macfarlane Burnet, the neoantigens on tumor cells can trigger an immune response. After the presentation of the tumor antigens, specific effector and memory cells are produced, in order to fight the tumor cells [3,4]. According to the immune surveillance theory, cancer cells go through three phases during their race with the host’s immune system: elimination, equilibrium, and escape phases. During the latter, they escape out of the immune system’s control [2]. This happens, because of the various defence mechanisms that cancer cells develop in order to avoid the immune response. When the immune system loses control over the cancer cells, it leads to their increased proliferation and tumor formation [3,4]. The main task of immunotherapy is to stimulate the patient’s immune system to attack cancer cells [1].

The beginnings of immunotherapy date back to 1893 when William Coley used live bacteria, and later, supernatants from bacterial cultures as a stimulator of the immune response in the treatment of cancer. Over the next few decades, scientists discovered the mechanisms that allow cancer to avoid the host’s immune response, and, thanks to this knowledge, research into this new treatment method was possible [5,6].

Tumors use various mechanisms to avoid the host response [7]. The most important ones include the upregulation of checkpoint receptor ligands, which results in the reduction of the number of tumor infiltrating lymphocytes (TIL), the production of soluble immunosuppressive factors (IL-10, TGF-beta), the downregulation of elements responsible for antigen presentation, and tumor infiltration by suppressor immune cells (regulatory T cells, Treg) [8].

Immunotherapy is a technique which can suppress, strengthen, or induct the immune system. It can be used to treat many diseases, including cancer. Anticancer immunotherapy works in an active, nonspecific manner (e.g., the administration of immunostimulating agents), an active, specific manner (e.g., administration of tumor antigens, DNA or RNA vaccines, dendritic cells vaccines), and a passive manner (e.g., administration of antibodies against tumor antigens, receptors or vascular growth factors, TILs or CAR-T cells, oncolytic viruses). A separate type of immunotherapy is the use of antibodies directed at the immune check points on immune cells and/or on cancer cells. Finally, the immune checkpoints blockade works by activating a tumor-specific immunological response. This method of immunotherapy is currently dominant in the treatment of cancer [9,10].

This review examines the relationship between the subjects’ diet and the effectiveness of immunotherapy in cancer patients.

## 2. Anti-PD-1, Anti-PD-L1 and Anti-CTLA-4 Immunotherapy—Mechanism of Action

The expression of immune checkpoints on cancer cells and immune cells plays an important role in tumor escape from immune surveillance. Programmed cell death protein-1 (PD-1) is a receptor found on T, B, NK, and myeloid-derived suppressor cells (MDSC) which is expressed in peripheral tissues in response to an inflammatory reaction. Biological function of the PD-1 receptor is to limit the activity of T CD4- and CD8-positive T cells and NK cells, which leads to suppressing the immune response and limits their lytic activity [11]. PD-1 has been shown to be upregulated on a significant portion of cancer infiltrating lymphocytes. Activation of PD-1 occurs through its interaction with programmed death ligand 1 (PD-L1), which is present on most cells, and is particularly highly expressed on immune cells, such as dendritic cells, and on neoplastic cells. Anti-PD-1 antibodies include nivolumab and pembrolizumab, whereas anti-PD-L1 antibodies include atezolizumab, durvalumab, and avelumab [11]. A PD-1 or PD-L1 blockade by immune checkpoint blockers (ICBs) enhances activation, expansion and function of T cells [8].

CTLA-4 (cytotoxic T cell antigen 4) is located on the surface of T lymphocytes, while its ligands—CD80 and CD86 molecules—are found on antigen presenting cells (APC). CTLA-4 is a negative modulator of the immune response in its early stages [12,13]. On the surface of T lymphocytes is expressed a CD28 molecule which, unlike CTLA-4, is a positive modulator of the immune response. It has the same ligands as CTLA-4, but it has less affinity to them. CD28 and CTLA-4 mutually control the level of the immune response [12,14]. Expression of CTLA-4 occurs also on Treg cells, causing their activation and immunosuppressive effect on cytotoxic T lymphocytes (CTLs). After the binding of CTLA-4 to CD80 or CD86, induction of 2,3-dioxygenase indoleamine (IDO) synthesis in dendritic cells and decreased production of IL-2 occur, both of which have strong T-cell inhibitory properties [13]. CTLA-4 blockade stimulates anti-tumor immunity of T cells and inhibits tumor growth [12,13]. Anti-CTLA-4 therapy uses monoclonal antibodies—ipilimumab and tremelimumab [15].

## 3. What Is Gut Microbiota?

The term “gut microbiota” is used to describe microorganisms inhabiting the human food tract. It includes bacteria, archaea, viruses, fungi, and protozoans [16], and is made by up to a 1000 species [17,18]. It plays a significant role in human health, participates in providing nutrients and vitamins, protects the body from pathogens, and modulates the function of the immune system [17,18,19,20,21,22,23,24,25,26,27,28,29]. It is also said to affect cancer patients’ response to immunotherapy [21,25,26,27,30,31,32,33,34,35,36,37,38,39] and affect mental health [15]. 

## 4. What Is the Composition of Gut Microbiota?

When talking about gut microbiota, three “enterotypes” are described. They are dominated by *Bacteroides*, *Prevotella*, or *Ruminococcus* [40,41]. The basis for their shaping is unknown; however, they seem to be independent of nationality, sex, age or body mass index [42]. The *Bacteroides*-dominated and *Bifidobacteriales*-dominated enterotypes are positively associated with a high-fat diet and high intake of animal protein, amino-acids and saturated fats and negatively associated with fiber intake. Enterotype dominated by *Prevotella* is associated with low values of all the above and high consumption of carbohydrates and simple sugars [41]. It has been reported that, among self-reported vegetarians, *Prevotella* enterotype is more common than others [40]. Subjects following a vegan diet have higher prevalence of the *F. prausnitzii* [43]. However, as can be seen in the descriptions below, there are conflicting reports about the involvement of different bacteria in gut microbiome in people who consume animal- or plant-based diets [40,41]. Provided information suggests that the subjects’ dietary patterns take part in the shaping of their gut microbiota on the Enterotype level [40,41,43].

## 5. What Kinds of Bacteria Can Be Found in Different Food Products?

According to the FAO/WHO definition from 2013, probiotics are live microorganisms which can have a beneficial effect on host’s health, when administered in correct amounts [44]. An important food group that could serve as a source of probiotics are fermented products. Fermented food products are products created with methods of controlled microbial growth, as well as enzymatic conversions of certain food components [45]. According to available data, some bacteria with probiotic properties (e.g., *Lactobacillus*, *Bifidobacterium*) could be isolated from certain food products [18,46]. Probiotic cultures of *Pediococcus* and *Lactobacilli* are often used in dairy products [47]. A study conducted on 1 *Pediococcus* and 9 *Lactobacillus* species demonstrated that the most acid resistant *Lactobacillus casei* strain survived in acidic environment for up to 63 days. This suggests the possibility of using probiotics in commercial pickles production [47]. Sauerkraut is believed to contain large amounts of organisms beneficial for human health [18,45].

Milk from farm animals may contain different strains belonging to bacterial species from the *Lactobacillus* (*Lb.*), *Enterococcus*, and *Pediococcus* genera. They contain 16 strains of *Lb. plantarum* and *Lb. fermentum*. A few strains of *S. thermophilus* and *Lb. plantarum* can be found in traditional Greek dairy products. The *Lb. plantarum* strain can also be found in traditional Polish cheeses. Tibetan kefir grain contains a strain of *Lb. kefiranofaciens*. Iranian Spar was found to contain *Lb. brevis*. Raw fermented meat products may contain strains belonging to the *Lactobacillus* or *Pediococcus* genera. *Enterococcus faecium* strain can be found in cooked meat products and in marine oysters. Fish and seafood contain various strains of species belonging to the *Lactobacillus* genera [18].

Pickled vegetables can be a source of bacteria strains belonging to various *Lactobacillus* species. Kimchi provides *Lactococcus lactis*, Korean fermented soybean paste provides strains belonging to *Enterococcus faecium* and a few other species from *Lactobacillales* order. Raw fruits and vegetables contain bacterial strains of the *Lactobacillus*, *Pediococcus*, and *Weisella* genera [18].

Although some of the fermented products undergo processing that deprives them of probiotic bacteria, there are still numerous fermented foods that do deliver significant amounts of probiotics (eg. kimchi, sauerkraut, various dairy products). During consumption of fermented food products, subjects ingest probiotic species that periodically enrich gut microbiota, which could have a possitive effect on human health [45]. Since gut microbiota affects the effectiveness of immunotherapy [21,25,26,27,30,31,32,33,34,35,36,37,38,39], consumption of fermented food products could be a relevant factor that, through gut microbiota alternation, influences patients’ response to the treatment.

## 6. How Does Diet Affect Intestinal Microbiota?

In order to determine the diet’s effect on patients’ response to immunotherapy, gut microbiota must be discussed. Patients may respond to immunotherapy differently, depending on the structure of their gut microbiota [19,20,27,48]. Intestinal microbiome can affect immunotherapy’s efficiency directly, through an interaction with the drug, or indirectly, through affecting the host’s natural immune system [19,20,40,49,50], thus modulating their response to the treatment [8]. Gut microbiota can also modify the side effects caused by the treatment [19]. An important factor influencing composition of the patient’s gut microbiota is their diet [20,40,41,42,49,51,52,53,54,55].

### 6.1. Animal-Based Diet Versus Plant-Based Diet

In the David et al.’s study, subjects were split into two groups—one with a diet based mainly animal products and one with a completely plant-based meal plan. Its authors showed a significantly stronger impact of an animal-based diet on the composition of gut microbiota. Increased amounts of *Provotella* in subjects with higher fiber intake within the last year were shown. Compared to a plant-based diet, an animal-based diet resulted in higher levels of amino-acid fermentation products and lower levels of carbohydrate fermentation products. The levels of aminoacid fermentation products correlated positively with the amounts of clusters comprised of putrefactive, bile-tolerant microbes (e.g., *Bacteroides* and *Clostridia*) and from saccharolytic microbes [41,42], and negatively with the numbers of beneficial bacteria, like *Bifidobacteria* and *Eubacteria* [41,54,56]. Moreover, a diet rich in high-saturated fatty acids also increases the amounts of anaerobic bacteria and *Bacteroides* [41,54,56,57]. It has also been shown that a high fat diet and an animal-based diet can promote growth of *Bilophila wadsworthia*—a bacteria producing hydrogen sulphide (H2S), suspected of inflaming intestinal tissue [41,42]. Furthermore, the animal-based diet increased the amount of microbial DNA and RNA responsible for coding sulfite reductase [42]. Western diet is high in animal protein, saturated fatty acids, and low in fiber [41,49,56].

Protein consumption increases the diversity of intestinal flora; however, the effects differ, depending on its source [54]. Whey and pea protein consumption increases levels of *Bifidobacterium* and *Lactobacillus*. It also limits the growth of *Bacteroides fragilis* and *Clostridium perfringens.* In addition to that, pea protein increases intestinal short chain fatty acids levels. An animal-protein-based diet stimulates bile-tolerant anaerobes’ (for example, *Bacteroides*) growth [54].

It seems that a diet rich in carbohydrates and fiber increases the variety and richness of intestinal microbiota [41,58]. A diet rich in carbohydrates increases *Bacteroidetes* amount. High fiber intake elevates *Bacteroidetes* levels and lowers *Firmicutes/Bacteroidetes* ratio [59,60] and *Bacteroides* (not to be confused with *Bacteroidetes*) levels [41]. Contradicting data was found regarding fiber’s influence on *Actinobacteria* levels [41,59,60]. 

At the same time, high fiber intake fosters *Firmicutes* and *Proteobacteria*, which are typically lower in subjects following a high fat diet [41]. High consumption of polyunsaturated fats fosters *Ruminococcus* growth inside the gut. A diet rich in carbohydrates and simple sugars leads to intensive growth of *Bacteroides* [56].

Vegetarian diet excludes meat and fish. It is rich in carbohydrates and fiber and can lead to an increased production of short chain fatty acids by gut bacteria [41,50,55], thus lowering local pH [41]. Changes in intestinal pH can strongly alter its microbiome. A one-unit pH decrease leads to decrement in *Bacteroides spp.* and stimulates the growth of butyrate-producing Gramm-positive bacteria. It also limits the growth of *Enterobacteriaceae*. Lower pH is well tolerated by the *Firmicutes spp.* and not tolerated by the *Bacteroides spp.* and *Bifidobacterium spp.* [41,43].

There has been a study, in which changes in the subjects’ intestinal microbiota were detectable within 24 h after switching to a certain diet; however, throughout the ten days of the study, the enterotype identity remained stable. This suggests that enterotype identity is defined by the long-term diet, rather than by short term changes in it [40,41,50,56,61]. However, it has been shown that already two weeks of an animal-based diet can lead to an increased level of *Fusobacterium nucleatum* in the subject’s gut microbiota [41,54,56]. On the other hand, there has been a three-month study, during which previously omnivorous people followed a vegetarian diet [50]. Their gut microbiota was checked before and after the three months period. There have been no significant changes on the phylum level; however, significant differences were noticed on the genera level. There was a decrease in the amounts of bile-tolerant organisms that are typical for an animal-based diet, and an increase in the amounts of species belonging to *Roseburia* and *Ruminococcus*, which are responsible for plant polysaccharides digestion [50]. Short-term vegetarian diet led to the prevalence of some probiotic species, including *Lb. plantarum* [50]. Long term vegetarians from the control group had higher levels of *Haemophilus*, *Neisseria*, *Aggregatibacter*, and *Veionella* [50].

The vegan diet excludes all animal products. It is associated with increased *Prevotella* numbers and higher prevalence of *F. prausnitzii*—a member of the *Firmicutes* [43] (Table 1).

### 6.2. Mediterranean Diet

The Mediterranean diet is a diet rich in vegetables, fruits, grains, nuts, and legumes. It is also characterized by high intake of unsaturated fats, especially olive oil, medium-high consumption of fish, moderate wine consumption, limited use of dairy products, and low intake of saturated fats, sweets, and meat [49,60]. It is associated with beneficial changes in gut microbiota [49] and increases the total amount of bacteria inside the gut [54]. In monkeys, long term Mediterranean diet led to increased levels of *Lactobacillus* in microbiome, compared to the Western diet [49,54]. There is a relationship between high adherence to the Mediterranean diet and increased levels of *Firmicutes* in the subjects’ gut microbiota [54]. Some reports state that, since the Mediterranean diet is rich in fiber, it promotes growth of short-chain fatty acids, producing *Bacteroidetes* and limits *Firmicutes* development [60].

### 6.3. Paleo Diet

The Paleo diet, also known as Paleolithic diet, is a diet mimicking the diet of humans of the Stone Age. It consists of high amounts of fruits, herbs, spices, and vegetables and moderate amounts of nuts and seeds. Meat, fish, and eggs consumption is on a moderate to high level. It excludes all processed foods, such as grains and legumes. Since the Paleo diet delivers a lot of carbohydrates accessible for gut microbiota, it is predicted to optimize its diversity [49]. The microbiome of Tanzanian Hadza hunter-gatherers, who consume a diet remarkably similar to the Paleolithic one, is more diverse, than the microbiome of urban Italians. It is dominated by *Firmicutes* and *Bacteroidetes.* It also contains more *Proteobacteria* and *Spirochaetes,* than the microbiome of urban Italians. However, when comparing these two, their evolutionary history must be considered, since it could affect the differences between their gut microbiota composition. In healthy Italians following the Paleo diet for more than one year, the microbiota consisted mainly of *Firmicutes* and *Bacteroidetes.* They were also found to have high levels of *Proteobacteria*, *Actinobacteria*, and *Verrucomicrobia.* The diversity of their intestinal microbiota was much higher than in Italians following the Mediterranean diet. It was comparable to the gut microbiome of Hadza hunter-gatherers. This shows that the loss of microbiome diversity among Western countries’ citizens could be counteracted [49].

### 6.4. Fasting

Fasting is a diet pattern in which a person restrains themselves from consuming solid food for a certain period of time. It was shown that every-other-day fasting leads to increased levels of *Firmicutes* inside the gut, while, at the same time, lowering the amounts of other phyla [49]. Different forms of fasting, such as intermittent fasting, multiday fasting, and diets mimicking fasting, improve intestinal microbiome’s diversity [57].

### 6.5. Carbohydrates and Artificial Sweeteners

A low-carb diet is a diet based on limited carbohydrates consumption. It is associated with weight loss and health improvement. It helps prevent hyperglycemia and hyperinsulinemia. In overweight patients, a low-carb diet with high protein intake lowers the amount of *Roseburia*, *Collinsella aerofaciens*, and *Enteroccocus rectale* [49,54]. 

A diet rich in complex carbohydrates increases the levels of *Bifidobacteria* and *Lactobacillus* [49,54,57], while simultaneously limiting the growth of *Enterobacteriacae* [48]. Excessive intake of refined sugars increases bile output, which triggers the proliferation of *Clostriudium difficile* and *Clostriudium perfringens* [49,54]. It is suspected that high sugar consumption leads to smaller diversity of species in gut microbiota. Replacing digestible carbohydrates with resistant starch in mice with pancreatic cancer modified their gut microbiota by shifting the balance towards the anti-inflammatory species. It reduced the intestinal levels of *Bacteroides acidifaciens*, *Escherichia coli*, *Ruminococcus gnavus*, and *Clostriduim cocleatum* and increased growth of butyrate-producing bacteria, for example, *Lachnospiraceae* [49]. It is also important to remember that excessive sugar or starch intake can lead to *Candida* overgrowth [57].

Artificial sweeteners used as natural sugar replacement are also suspected to induce changes in intestinal flora [54,62]. It was observed that saccharin-fed mice had intestinal dysbiosis, with relatively increased level of *Bacteroides* and reduced amount of *Lactobacillus reuteri*, which is the opposite of the changes induced by natural sugars [54] (Table 2).

### 6.6. Ketogenic Diet

The ketogenic diet is a low-carbohydrate diet that reduces their amount to the point at which a low insulin level and an elevated cortisol level induce the production of ketone bodies [49]. The aim of this diet is to mimic a fasting state by making fat—instead of carbohydrates—a dominant caloric source [63]. It is known to promote metabolic health and prevent cancer; however, its effect on gut microbiota is not well-known. In a mouse model of autism, it normalized levels of *Akkermansia muciniphila* (*A. muciniphila*) and increased the *Firmicutes*/*Bacteroides* ratio, which is typically low in patients with autism spectrum disorder [49]. In healthy mice a ketogenic diet had a beneficial effect on neurovascular function, increased the amount of *A. muciniphila* and *Lactobacillus* and lowered the amount of pro-inflammatory bacterial taxa, like *Desulfovibrio* and *Turicibacter*. At the same time, it lowered the overall microbial diversity. This effect of the ketogenic diet comes from the low content of polysaccharide, which is the main energy source for many gut bacteria [63]. In patients with multiple sclerosis, within the first few weeks, a ketogenic diet lowered the total intestinal bacteria concentration; however, when held up for 6 months, it was able to restore the microbial mass to levels similar to subjects in healthy controls [49,63]. In patients with refractory epilepsy, the ketogenic diet, which is considered an effective treatment in patients with drug resistance, increases levels of *Bacteroides* and *Prevotella* and lowers the amount of *Cronobacter* [49,57,63]. Children with refractory epilepsy, after one week and after 6 months of a ketogenic diet, had increased levels of *Bacteroidetes* and decreased levels of *Proteobacteria* and *Firmicutes* (which is in contradiction with the results obtained in the mouse model). This indicates that a ketogenic diet can rapidly alter microbiota in children by shifting the balance towards beneficial bacteria. When put together, this data suggests that a ketogenic diet can reverse dysbiosis associated with neurological disorders [49].

### 6.7. Gluten-Free Diet

A gluten-free diet is a diet that excludes all sources of gluten, for example, wheat and rye. In patients with Crohn’s disease who followed a gluten-free diet for at least two years, it has been shown to lower the amount of *Escherichia coli* and *Staphylococcus*, and have no effect on beneficial species, like *Bifidobacterium* and *Lactobacillus* [63]. It is suspected that these properties of the gluten-free diet come from the reduction of fructans with prebiotic properties—they foster beneficial butyrate-producing bacteria (for example, *Faevalibacterium prasunitzii*) and, at the same time, lower the amount of *Bacteroides* and *Clostridium* [41]. However, other studies suggest that, in patients with Crohn’s disease, a gluten-free diet can lead to decreased amounts of beneficial bacteria, like *Lactobacillus*, *Enterococcus*, and *Bifidobacteria*, and stimulate the growth of harmful species, such as *Bacteroides*, *Staphylococcus*, *Salmonella*, *Shigella*, and *Klebsiella* [63]. In healthy subjects, a gluten-free diet lowered the levels of beneficial bacteria, while simultaneously increasing the amount of *Enterobacteriaceae*, which are responsible for gut inflammation [54,63].

### 6.8. Low-FODMAP Diet

A low-FODMAP (fermentable oligosaccharides, disaccharides, monosaccharides, and polyols) diet is a diet considered to be beneficial to patients with inflammatory bowel syndrome (IBS) [57,63]. A four-week dietary intervention in patients with IBS showed that restricting carbohydrates according to the low-FODMAP diet led to a reduction of *Bifidobacteria* concentration. Total bacteria levels, as well as the amounts of specific bacteria, like *Bacteroides*, *Clostridium coccoides*, *Enterococcus*, *Eubacterium rectale*, *Faecalibacter prausnitzii*, *Lactobacillus*, and *Prevotella*, were not affected by the intervention. However, in another study, subjects who followed a low-FODMAP diet for three weeks, besides a decreased amount of *Bifidobacteria*, showed elevated levels of *Actinobacteria* and *Lachnospiraceae*, as well as generally increased diversity, when compared to subjects who followed a high-FODMAP diet. Furthermore, the subjects following a high-FODMAP diet had decreased levels of *Firmicutes*, *Clostridiales*, and overall microbiotic diversity. On the other hand, in a small uncontrolled study performed in children with a low-FODMAP diet for one week, no microbiome changes were reported [63]. Patients with saccharolytic metabolic capacity of microbiota may experience benefits from a low-FODMAP diet. Long-term use of low-FODMAP diet is problematic, due to the limited amount of healthy plant foods and natural probiotics [63].

This suggests that whether dietary interventions can alter the subject’s gut microbiota depends on the exact composition and duration of the diet.

## 7. The Microbiome and the Immune System

There is a symbiosis between the development and regulation of the innate and adaptive immune system and the gut microbiota [21,24,64,65,66,67,68]. The immune system is required to provide a proper balance between the microbiota, oral food antigens tolerance and the surveillance against infectious factors [21,64,65,66,69]. The host immune system influences the composition and morphology of gut microbiota [21,64,65]. The gut microbiome contributes to the development of the distant lymphoid tissues, such as peripheral lymph nodes or the spleen [25,70,71]. It regulates lymphocyte subpopulations in secondary immune organs and helps regulate the immune system regarding local mucosal immunity [21,64,65,70,72]. Gut microbiota promotes an anti-cancer host immunity, maintains bacterial diversity and prevents colonization of the gastrointestinal tract by pathogens [25,70]. This balance is the result of the co-operative crosstalk between the host’s lymphoid structures, epithelial cells, and intestinal microbiota [21,64,65,70,72]. Therefore, the interaction between gut bacteria and the host’s immune system is bidirectional [23,26,56].

The microbiome interacts with the host through multiple mechanisms. Pathogen-associated molecular patterns (PAMPs), such as flagellin, lipopolysaccharide, and peptidoglycan, are recognized by the immune cell pattern recognition receptors (PRRs), e.g., toll-like receptors (TLRs), on the leucocytes and epithelial cells, which moderate the reaction between the host’s immune system and bacteria [21,26,64,69]. Immune cells containing a large number of PRRs are found at the microbiota cluster’s surface [73]. PRRs activate a cascade of intracellular signaling pathways within the immune system cells which recognize pathogens and boost B- and T-cell related response [23,26].

Gut bacteria impact the differentiation of naive T cells in the mesenteric lymph nodes. PAMPs might induce maturation of antigen-presenting cells, like dendritic cells (DCs). Dendritic cells interact and stimulate naive T cells and their differentiation to T helper (Th) and Treg cells. DCs and Th cells can directly stimulate CD8-positive T lymphocytes [21,26,64,69]. In addition to influencing local immunity, the microbiota can also modulate systemic immune responses through immune cell priming. Dendritic cells and other innate immune effectors are activated through toll-like receptors and might produce cytokines and interferons that act both as paracrine and endocrine factors at distant sites [21]. There are reports in scientific literature about an increased level of interferon α/β signaling in lung stromal cells by intestinal bacteria [71].

Many microbial ligands can stimulate the activation of nuclear factor kappa B (NFκB) in leucocytes, and the production of TNF-α and IL-1. The presence of the gut microbiota results in expression of the anti-microbial peptide, regenerating islet-derived 3 gamma (Reg III-γ) in Paneth cells. Other studies discovered that these antimicrobial particles are the key mediators of homeostatic balance between the intestine microbiota and the host’s immune defense against enteric pathogens [64].

A key role is also played by Treg lymphocytes that down-regulate the pro-inflammatory response leading to immune-tolerance and immunosuppression. It can be achieved through the production of IL-10, TGF-β, IL-35, IL-2, and more [20,64]. Some gut bacteria can secrete anti-inflammatory mediators (thymic stromal lymphopoietin, TGF-β, IL-10, IL-25, and IL-33). It is considered that certain bacterial species can drive Treg maturation and TGF-β production through alternative pathways dependent on polysaccharide A (PSA) binding to TLR2 on dendritic cells [21]. Research showed that the processes of differentiation of T cells into Treg cells and their functional maturation take place in the intestine where the commensal microbiome is present, rather than in the thymus [64]. IL-10 is responsible both for maintaining homeostasis in the colon and for the suppression of the inappropriate activation of myeloid cells, γδ T cells, and Th17 cells [64].

The research showed that *Bacteroides fragilis* and *Eshcerichia coli*, when enriched by *Firmicutes* (mainly *Ruminococcaceae* or *Lachnospiraceae*), as well as *Proteobacteria* and various species of *Bacteroidales,* stimulate T cell migration into colorectal carcinoma tissues and increase expression of T cell-recruiting chemokines. *Fusobacteria* speaks for poor prognosis in colorectal carcinoma, probably because of the inhibition of tumor-infiltrating lymphocytes and the inhibition of NK cells [67]. 

Gut microbiome is responsible for correct programming of the Th1/Th2 balance, the lack of which results in a tendency to Th2-type predomination and allergic responses [69]. *Bacteroides fragilis* produces PSA, which corrects Th cells deficiency and Th1/Th2 imbalance in germ-free mice [65,74]. *Bernesiella intestinihominis* enhances cytotoxic T cell (CTLs) and Th1 response of the entire immune system. *Escherichia coli* and *Escherichia coli Nissle (EcN*) activate TLR and up-regulate the production of pro-inflammatory cytokines (IL-6, IL-8, and IL-1β [22]. *Bilophila* promotes pro-inflammatory Th1 immunity [54], whereas *E. hirae* induces Th17 cells’ response and has the potential to increase the cytotoxic T cells/Treg cells ratio [65]. The adaptive immune system can be activated by the nucleotide-binding oligomerization domain 2 (NOD2). NOD2 is a component of the bacterial cell wall. This component facilitates boosting the production of α-defensin or other regulatory particles, such as IFN [75].

Bacteroides, Lactobacillus acidophilus [22] Lactobacillus murinus, Lactobacillus reuteri, Helicobacter hepaticus, and some strains of Clostridia [64,65] and Faecalibacterium might enhance the expansion of Treg cells or stimulate the production of anti-inflammatory cytokines [65], which leads to the suppression of the host’s immunological response [64,65]. Faecalibacterium prausnitzii, Eubacterium, Roseburia, Bifidobacterium longum Ruminococcus, Alistipes, and Lactobacillus produce short-chain fatty acids (SCFAs) [54]. SCFAs inhibit T cells and stimulate Treg cells [50]. Microbial SCFAs and dietary fiber fermentation products may stimulate myeloid dendritic cells population in the bone marrow. They can also stimulate the phagocytic capacity of these cells [70]. Higher SCFAs levels inhibit the expression of the pro-inflammatory cytokine tumor necrosis factor (TNF) in mononuclear cells and in neutrophils [50], and can lead to an inactivation of NF-κB [26]. SCFAs may also regulate the host’s immunity through the inhibition of histone deacetylase activity [21,26,69].

Intestinal epithelial cells (IECs) may respond to various types of cytokines and chemokines, and express particles interacting with lymphocytes. They can also secrete antimicrobial peptides (AMPs) or mucins. IECs express CD1d, an MHC-like molecule, which presents glycolipids to T cells and NK (natural killer) cells and, after the activation of STAT3, IECs produce the anti-inflammatory IL-10. *Bacteroides thetaiotaomicron* stimulates IECs to produce C-type lectin, AMPs, α- and β-defensins, and many hydrolytic enzymes. *B. thetaoiotaomicron* increases the expression of matrix metalloproteinases (MMP), which are required to activate defensins. *Bacteroides thetaiotaomicron* might down-regulate an inflammatory response of the immune system by interfering with the activation of NFκB in the peroxisome proliferator-activated receptor-γ (PPARγ)-dependent pathway [56].

Intestinal microbiota intensifies the capacity of mucosal B lymphocytes to produce IgM and IgA antibodies. SCFAs might have an influence on a local immunity via stimulation of IgA production by plasmocytes [21,26,69]. Polyamines (the products of metabolism of the gut microbiota), such as putrescine, spermine, and spermidine, can stimulate the secretion of IgA in the intestine, as well [26]. While the IgA is lacking, anaerobic bacteria expand, including segmented filamentous bacteria (SFB). It is considered that SFB support the population of inflammatory Th1 and Th17 cells, and IFN-γ production [40]. The Th17 cells produce IL-17, which can stimulate IECs to secrete anti-microbial proteins and form epithelial tight junctions [21,64,69]. The Th17 lymphocytes are functionally plastic because they are able to alter the release of particular cytokines on the basis of local inflammatory conditions [13,21,69]. *Enterococcus faecium* is an example of a bacterium showing a positive correlation with IgA levels, whereas *Clostridium ramosum*, *Eggerthella lenta*, *Lactobacillus casei*, *Leuconostoc mesenteroides*, and *Bacteroides uniformis* show a negative correlation with the level of IgA in the intestine [50]. IgA blocks the adhesion of the bacteria to epithelial cells, and affects bacterial virulence [69].

The immune system also includes innate lymphoid cells (ILC). The main place of their occurrence is the intestinal and respiratory mucosa. They are characterized by a rapid immune response and the absence of an antigen-specific receptor. There are 5 subgroups of ILC: ILC1, ILC2, ILC3, lymphoid tissue inducer cells (LTi), and NK cells. Mice with the absence of ILC3 showed increased numbers of segmented filamentous bacteria (SFB) and *Clostridiales* species. On the other hand, the expression of the inhibitor of DNA-binding 2 (Id2) in ILC3 influences early resistance to *Citrobacter rodentium* colonization [68].

The balance of the gut microbiota (eubiosis) is crucial in maintaining proper immunity. Damage of the mucosal barrier impairs the immune response of the host. The gut bacteria translocate to the mesenteric lymph nodes and enter the peripheral circulation, which might lead to local inflammation or the inflammation of the whole organism [69]. Especially in older people, high levels of gram-negative bacteria in the gut are often responsible for chronic inflammation [76]. In turn, dysbiosis caused by antibiotics or an incorrect diet leads to the inactivity of the immune system and the development of various diseases.

## 8. The Impact of Microbiome on the Efficacy of the Immunotherapy

Data shows that gut microbiota has a strong influence on the efficacy of cancer immunotherapy, chemotherapy, and radiotherapy [77]. Monoclonal antibodies, such as anti-PD-1/PD-L1 or anti-CTLA-4, are successfully used in cancer immunotherapy [24,31,78,79]. There are many reports stating that microbiome has a significant role in enhancing or reducing the efficacy of immunotherapy [80] and disturbance of the physiological gut microbiome may lead to primary resistance to immune checkpoint therapy [32,80,81]. A better response is observed in the presence of specific gut microbes, while antibiotic treatment is associated with poor response to anti-PD-1, anti-PD-L1 or anti-CTLA-4 immunotherapy [16,19,20,26,32,33,64,67,69,80,81,82,83,84,85,86,87,88]. Patients without antibiotic treatment or with short-term exposure to antibiotics (<7 days) had longer overall survival and progression-free survival than patients with longer antibiotic exposure [83]. The influence of antibiotic administration 60 days before the treatment is not as strong as its influence within 30 days prior to the immune checkpoint blockade treatment [86]. It is caused by a decrease in the gut microbiome’s variety and richnes leading to lower pro-inflammatory cytokine production and less pronounced tumor necrosis [31,39]. However, one study showed that antibiotic administration did not influence the response rate and PFS in NSCLC patients treated with nivolumab [80]. One should take into consideration that patients receiving antibiotics are in a worse general condition, and this may have an impact on the treatment outcome [88].

*Bacteroides thetaiotaomicron* and *Bacteroides fragilis* improve response to the anti-CTLA-4 therapy [34,80,89]. Patients with higher abundance of that kind of *Bacteroides* in the gut microbiome had decreased numbers of the regulatory T cells (Treg) and myeloid derived suppressor cells (MDSC) [30], and they had an increased number of Th1 cells [85]. In mice having *Bacteroides* (mainly *B. fragilis*) and *Burkholderia* in the guts, different cancers displayed slower growth, and the mice experienced reduced immunotherapy-induced intestinal epithelial cell necrosis, when they were treated with the anti-CTLA-4 antibodies [20,24,27,31,35,88,89,90,91]. 

*Bacteroides* was reported to enhance the anti-CTLA-4 therapy’s efficiency and restore the its effects after an administration of antibiotics through a proposed mechanism involving the activation of the IL-12-dependent Th1 cells with cross-reactivity to tumor and bacterial antigens. Other mechanisms trigger dendritic cell maturation [32,65,67,69,70,72,83,90,92]. This effect can be achieved through giving *B. fragilis* to mice orally, transferring *B. fragilis*-specific T lymphocytes or immunization with polysaccharides of the *B. fragilis* species.

The MCA205 sarcoma in mice shrank during the anti-CTLA-4 therapy, when *Bacteroides fragilis*, *Bacteroides thetaiotamicron*, and *Burkholderia* species were present in their organisms. However, it is considered that *Escherichia coli* or other common *Bacteroides* species, like *Parabacteroides distasonis* or *Bacteroides uniformis*, do not exert an influence on anti-CTLA-4 treatment’s efficacy [84].

According to the Lukas F. Mager et al.’s experiment, in mice models into which M38 colorectal cancer cells were transplanted, the monocolonization with the *Olsenella* species, *Bifidobacterium pseudolongum*, and *Lactobacillus johnsonii* significantly strengthened the anti-CTLA-4 treatment’s effect in comparison to germ free models or models monocolonized with *Prevotella* or *Colidextribacter* species. Notably, an isolated strain of *B. pseudolongum* improved the efficiency of anti-PD-L1 therapy, as well [93].

After an administration of the anti-CTLA-4 therapy, notable changes in the composition of the gut microbiota in mice were spotted, including an increase in *Burkholderiales* and *Bacteroidales* and a decrease in *Clostridiales* [21]. It is supposed that *Burkholderiales* and *Bacteroidales* can affect IL-12-dependent Th1 immune response, which facilitates better disease control [90].

Eight species (Bifidobacterium adolescentis, Enterococcus faecium, Collinsella aerofaciens, Bifidobacterium longum, Klebsiella pneumoniae, Veillonella parvula, Lactobacillus, and Parabacteroides merdae) were more abundant in the microbiome of the patients suffering from unresectable melanoma, non-small cell lung cancer, urothelial cancer, or renal cell carcinoma, who responded to anti-PD-1 therapy. Two species were more abundant in a group with poor response: Ruminococcus obeum and Roseburia intestinalis [25,94].

The abundance of *Akkermansia muciniphila* correlated with a better response to immunotherapy in lung carcinoma [93], renal cell carcinoma [72,93], and urothelial carcinoma patients [72]. In renal cell carcinoma, the presence of *Alistipes* in gut microbiome was linked to a better clinical outcome, as well [81].

The high proportion of the following species among microbiota increases the effectiveness of immunotherapy: Akkermansia muciniphila, Alistipes indistinctus, Bifidobacterium breve, Propionibacterium acnes, Prevotella copri, Rikenellaceae, Staphylococcus aureus, Streptococcus, Peptostreptococcus, Oscillospira, Faecalibacterium prausnitzi, Bacteroides plebeius, Enterobacteriaceae, and Enterococcus hirae. Sutterella, Ruminococcus bromii, and Dialister were less abundant in NSCLC patients with a good response to anti-PD-1 therapy [73,76,81]. In patients with a poor response, enrichment of Ruminococcus unclassified was spotted in their gut [76].

In another study, patients suffering from metastatic melanoma, receiving ipilimumab with a high level of *Faecalibacterium*, and, surprisingly, *Ruminococcaceae* gained a better response to anti-PD-1 treatment [20,27,31,69,80,83,86,91,94,95]. *Faecalibacterium* and *Ruminococcaceae* improved effector T lymphocytes’ function in the periphery, and, in the tumor microenvironment, they also increased antigen presentation by APC [67]. *Bifidobacterium longum*, *Collinsella aerofaciens*, and *Enterococcus faecium* also improve anti-PD-1 response in metastatic melanoma patients [66,86,87]. *Clostridium XIVa*, *Gemmiger, Bacteroides thetaiotaomicron*, and *Escherichia coli* in feces (but not in oral microbiome) often correlated with a poor clinical outcome [19,94,96].

In subsequent studies, it was noted that *Bifidobacterium longum, Collinsella aerofaciens* and *Enterococcus faecium* were overrepresented in the anti-PD-1 immunotherapy responders with metastatic melanoma before treatment [27,65,73]. Ipilimumab and nivolumab responders were colonized with *Bacteroides thetaiotamicron*, *Holdemania filiformis*, and *Faecalibacterium prausnitzii*, while pembrolizumab responders were colonized with *Dorea formicogenerans*. All immune checkpoint therapy responders had a significant amount of *Bacteroides caccae* [80,97].

The gut microbiome may have had a key influence on the response in hepatocellular cancer patients treated with anti-PD-1 immunotherapy. Patients with hepatocellular carcinoma treated with anti-PD-1 antibodies had better outcomes if their intestinal microbiota contained the following bacterial species: *Streptococcus thermophilus*, *Fusobacterium ulcerans*, unclassified *Candidatus Liberibacter*, *Lactobacillus mucosae*, *Ruminococcus obeum*, unclassified *Lachnospiracae*, *Ruminococcus bromii*, unclassified *Subdoligranulum*, *Bacteroides cellulosyticus*, *Lactobacillus gasseri*, *Anaerotruncus colihominis*, *Eubacterium hallii*, *Dorea formicigenerans*, *Lactobacillus vaginalis*, *Dalister invisus*, *Lactobacillus oris*, *Akkermansia muciniphila*, *Bifidobacterium dentium*, *Megasphera micronuciformis*, and *Coproccus comes*. Worse clinical outcome was noted in patients with the following bacterial species in their gut: *Bacteroides nordii*, *Fusobacterium varium*, *Bacteroides eggerthii*, *Veillonella dispar*, *Bacteroides uniformis*, *Veillonella atypica*, *Lactobacillus salivarius*, *Enterobacter aerogenes*, *Bifidobacterium bifidum*, *Aggregatibacter aphrophilus*, *Haemophilus pittmaniae*, *Bacteroides fluxus*, *Escherichia albertii*, *Bifidobacterium adolescentis*, and *Megasphaera elsdenii* [36].

Th cells and CTLs produce IFN-γ in response to *Akkermansia muciniphila* alone [27,67,69] or when it is combined with *Enterococcus hirae* [69]. The production of IFNγ by Th cells is also stimulated by: *Bacteroides uniformis*, *Bacteroides dorei*, *Ruthenibacterium lactatiformans*, *Eubacterium limosum*, *Paraprevotella xylaniphila*, *Parabacteroides distasonis*, *Parabacteroides gordonii*, *Alistipes senegalensis*, *Parabacteroides johnsonii*, *Fusobacterium ulcerans*, and *Phascolarctobacterium succinatutens* [85]. This is linked to prolonged progression-free survival in patients treated with the anti-PD-1 immunotherapy [27,67,69].

**Figure 1:**
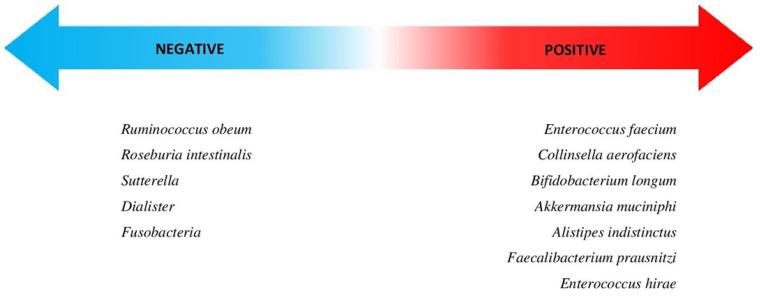
The influence of selected gut microbiome on anti-PD-1/anti-PD-L1 therapy [25,27,36,65,67,69,73,76,81,94].

*Bifidobacterium* increases the number of dendritic cells, expression of MHC (major histocompatibility complex) and CTLs activation. These mechanisms can enhance therapeutic effects of antibodies targeting the PD-1 or PD-L1 in *B16 melanoma*-bearing mice [78,98]. Oral administration of *Bifidobacterium* species promotes this response by increasing the level of the tumor-specific T-cell response, the tumor infiltrating by CTLs and increased IFN-γ production, enhancing CTLs priming and improving the anti-PD-L1 treatment’s efficiency [19,21,70,72,73,80,92] (Figure 1).

There are many immune-related adverse events caused by the anti-CTLA-4 antibodies, including colitis [21,24,31] and inflammatory bowel disease [21,31]. Anti-CTLA-4 toxicity can be induced by the gut bacterial composition. Certain bacterial species, including *Rikenellaceae*, *Barnesiellaceae*, *Bacteroidaceae*, *Lactococcus*, *Bifidobacterium animalis*, *Burkholderia cepacia*, *Faecalibacterium prausnitzii*, *Helicobacter hepaticus*, *Lactobacillus*, and *Clostridia*, are essential for maintaining a tolerogenic state in the mucosa. They are linked to the resistance to checkpoint-blockade-induced colitis. It is reached by inducing the production of anti-inflammatory cytokines, the production of nitric oxide, shifting the Th1/Th2 balance and inducing Treg cells differentiation. It is assumed that this particular effect can be reached by the modulating polyamine transport and synthesis of group B vitamins by these bacterial species [19,21,31,32,56,64,66,69,72,92]. Research showed that patients with a higher level of *Faecalibacterium prausnitzii* and other related *Firmicutes*, and with a lower level of *Bacteroidetes*, had a higher risk of colitis caused by the anti-CTLA-4 therapy [21]. Moreover, colitis might affect anti-cancer efficacy of the CTLA-4 blockade [90].

Adverse effects of the anti-PD-1/PD-L1 treatment include pneumonitis, thyroid dysfunctions [72], and colitis with diarrhea. Data shows that *Bacteroides*, *Parabacteroides*, and *Firmicutes* were more abundant in the gut of patients without diarrhea. *Veillonella* was less abundant in this kind of patients [79].

In mice tumor models (including colon cancer), the use of antibodies against the IL-10 receptor (anti-IL-10R) combined with CpG-oligodeoxynucleotides (ODN), which is a ligand for TLR9, is linked to stimulation of macrophages, tumor-associated myeloid monocytes, DCs, and to release of TNF-α, CXCL-10, IL-12, and IL-1. They give a clinical benefit of extending OS and reducing tumor volume, and inducing hemorrhagic tumor necrosis. Interestingly, they are ineffective when the mice are treated with antibiotics or when the physiological bacteria are not present in their gut [24,92]. Transfer of *Ruminococci* or *Alistipes shahii* restores the efficacy of this kind of treatment because these bacteria species are correlated with an increased TNF-α production, whereas *Lactobacillus* species are negatively correlated with TNF-α production and worsen the clinical outcome [16,24,72,84,99].

In connection with these discoveries, the use of FMT (fecal microbiota transplant) is considered as a way to enhance the effectiveness of the immunotherapy. Efficacy of this therapy seems to be related to a higher amount of IFN-γ, anti-tumor T helper, and cytotoxic cells, intratumoral mature DCs, and lower levels of intratumoral Treg cells. However, there is a risk that bacteria might contribute to inflammation-induced carcinogenesis; therefore, this method has to be used cautiously [19,31,34,82,84,92]. 

Oral application of *Akkermansia muciniphila* in FMT to non-responders reconstructed the antitumor effect of anti-PD-1 treatment through the accumulation of CCR9+ CXCR3+ CD4+ T cells [81]. While *Enterococcus hirae*, *Akkermansia muciniphila*, or *Alistipes* probiotics were substituted for the FMT, tumor growth was reduced by 40%, in comparison to the immune checkpoint therapy without probiotics for the RET melanoma, MCA205 sarcoma, and Lewis lung carcinoma models [84].

Prebiotics, including chemical or dietary ones, facilitate the colonization and the relative expansion of the chosen bacteria species in the gut, which may boost an anti-tumor immunity [70]. Probiotics are essential to restore anticancer adaptive T-cell response. It is considered that *Bifidobacterium* and *Burkholderiales* may play a role of “anti-cancer probiotics”. The distribution of *Bifidobacterium species* in the mucosal epithelium of the intestine and its connection with *Burkholderiales species* is recognized through the pyrin-caspase-1 inflammasome and synergizing through TLR2 and TLR4 signaling pathways [91]. Because of their impact on cancer immunotherapy, the FDA has indicated that probiotics should be developed and regulated as a drug [34,82,90]. 

## 9. Influence of Other Dietary Factors on the Immune System and the Effectiveness of Immunotherapy

### 9.1. Vitamin D

Vitamin D, in addition to its classic effects on the skeletal and muscular-nervous system, has a significant effect on the immune system [100]. Vitamin D plays a role in the activation of the immune system, mainly T cells. It stimulates the innate immune response through monocytes and macrophages by enhancing their phagocytic and chemotactic response. Vitamin D increases the synthesis of prostaglandin E2, as well as natural antibacterial substances (cathelicidin). It also inhibits the maturation and differentiation of dendritic cells [101]. Studies carried out on T cell cultures showed that when vitamin D was added, there was a significant increase in PD-1 expression on these cells. In addition, the expression of CTLA-4 on these cells is increased [102,103,104]. The same studies noted a significant decrease in IFNγ synthesis under the influence of calcitriol, while IL-4 production was increased. The direct effect of vitamin D on the Th1 and Th17 cell response includes a decrease in cytokine synthesis and an increase in the cytokine production by Th2 cells. Vitamin D also promotes the development of Treg cells. Deficiency of vitamin D or its receptor (VDR) shifts the response towards the Th1 lymphocyte [102].

Due to its effect on T cells, vitamin D may affect the efficacy of the immune checkpoint inhibitors therapy [105]. Moreover, vitamin D deficiency can often be seen in cancer patients. In addition, some vitamin deficiencies are more likely in people who follow a vegetarian, vegan, or fruit-based diet; therefore, supplementation is recommended in these cases [56]. Research shows that vitamin D affects cancer immunotherapy, for example, 25-hydroxyvitamin D affects nivolumab concentration [100]. 25-hydroxyvitamin D concentration is influenced by the polymorphism of the gene encoding of the vitamin D binding protein (VDBP). The occurrence of the rs7041 allele is associated with lower 25-hydroxyvitamin D concentrations. Assessment of the 25-hydroxyvitamin D level and rs7041 *VDBP* genotype before and during nivolumab treatment allows for adequate supplementation of vitamin D to achieve better treatment results, by reducing the risk of cancer progression. Higher risk of more severe adverse events has been observed in patients diagnosed with vitamin D deficiency before starting immunotherapy [100]. Studies on patients with advanced metastatic melanoma have shown that both a low initial value and poor supplementation of vitamin D are associated with a worse prognosis [105].

Numerous reports inform that vitamin D affects the intestinal microbiome. The Luthold et al. research showed that low a level of vitamin D is correlated with an increased amount of *Haemophilus* and *Veillonella*. On the other hand, a high level of vitamin D increases the amount of *Prevotella* [106].

### 9.2. Vitamin B1

B cells in the intestine are transformed into IgA-synthesizing plasma cells. Naive B lymphocytes use the tricarboxylic acid cycle (TCA), while IgA-producing plasma cells use both the TCA pathway and glycolysis for energy production. A diet without vitamin B1, which is involved in the TCA pathway, leads to a decrease in the amount of the naive B lymphocytes in the gastrointestinal tract, but it does not reduce the IgA-producing plasmocytes’ levels. Vitamin B is needed to maintain an IgA-dependent response to oral antigens. A reduction in pre- and pro-B cells in the bone marrow was observed in studies on mice with a vitamin B deficient diet [107].

### 9.3. Obesity

With more and more people suffering from it, obesity is now a global problem. It is associated with an increased incidence of neoplasms, worse prognosis, and increased mortality. It is caused by metabolic and inflammatory changes that occur in adipose tissue [108]. In 2016, about 13% of the world’s adult population (11% of men and 15% of women) was obese, with over 340 million children and adolescents aged 5–19 being overweight or obese [109]. In people with normal body mass index (BMI = 18.5–24.9), the adipose tissue microenvironment (ATME) is rich in anti-inflammatory cytokines. This situation changes in the direction of inflammation as weight increases. Then, the production and release of pro-inflammatory cytokines, such as TNFα, IFNγ, IL-1β, and IL-6, into ATMEs are increased. Chronic fibrosis and vascular inflammation also occur [110]. 

Many substances formed in ATME affect the biology of cancer cells. Mast cells produce cathepsin S, which may cause resistance to cytotoxic drugs. Remodeling of the extracellular matrix and an increase in the production of vascular endothelial growth factor by adipose tissue stromal cells and myofibroblasts facilitates angiogenesis of the tumor. Newly formed vessels and other changes in ATME stimulate the innate immune response. Macrophages form crown-like structures (CLS), produce a large number of cytokines, and cause desmoplasia. T cell activation is suppressed by the PD-1 activation [110]. Naturally occurring thymus involution is accelerated in obesity due to excessive fat accumulation. This reduces the number of new naive T cells. The function of B lymphocytes that are dependent on T lymphocytes is partially impaired and the production of antigen specific IgG is reduced [90]. In obesity, we may observe increased amount of free fatty acids (FFA) [111]. In contrast, polyunsaturated fatty acids (PUFA), such as α-linolenic acid (n-3 PUFA) and linolenic acid (n-6 PUFA), have anti-inflammatory properties. The anti-inflammatory effect of PUFA is manifested by their effect on bone marrow suppressor cells [75,112].

In cancer patients, particularly with breast or prostate cancer, the presence of CLS is associated with worse treatment outcomes and leads to tumor progression. Research indicates that CLS may be used as a prognostic biomarker in some cancers. In breast cancer models, it has been shown that PD-L1 expression is upregulated in myeloid-derived suppressor cells, which can lead to the escape of the tumor from immune surveillance [108,110]. After blocking PD-1 with a monoclonal antibody—nivolumab—, T cells regain a strong effector function, hence the high effectiveness of checkpoint inhibitors in obese patients with melanoma [110,113]. A recent study has shown that obesity induces T cell dysfunction and an upregulation of PD-1 on T cells, which is partially mediated by leptin. However, the polarization of T cells towards an exhaustive phenotype is correlated with improved response rates to anti-PD-1 therapy in the setting of obesity. Therefore, obesity, which is associated with T cell dysfunction, also paradoxically induces a better response to anti-PD-1/PD-L1 immunotherapy [114,115,116].

On the other hand, studies in mice showed that reduced leptin levels could enhance the effectiveness of checkpoint inhibitors. Treatment results were better among the group of mice with obesity induced by a leptin deficiency, than in mice with diet-induced obesity and without leptin deficiency. Application of a recombinant leptin receptor to DIO (diet-induced obese) mice caused a decrease in leptin levels, which was followed by an increase in the effectiveness of the immunotherapy. In DIO mouse models, lower numbers of functional dendritic cells and a reduced tumor invasion by CTLs were responsible for the reduced effectiveness of immunotherapy [117].

Dietary supplementation with PUFAs may result in increased death of cancer cells by altering the composition of the cell membrane and thereby increasing the sensitivity to lipid peroxidation [111]. This has a positive effect on immunotherapy. An increase in fatty acid oxidation (FAO) in peripheral blood MDCSs occurs in patients with various types of cancer. Pharmacological blocking of FAO in combination with immunotherapy or chemotherapy blocks the immunosuppressive properties of MDSC [112].

Obesity is usually caused by inappropriate dietary habits and consuming too many calories in relation to the organism’s demand [108,109,118]. It is related to the consumption of foods rich in carbohydrates and fats, as well as limited consumption of vegetables [109,118]. Obesity leads to predominance of *Firmicutes* over *Bacteroidetes*. After weight loss, the number of *Bacteroidetes* increases [119].

### 9.4. Salt Level in the Diet

Studies in mice have shown that high salt intake can inhibit tumor growth, and it also can affect T cells and suppress myleoid-derived suppressor cells. This mechanism leads to better anti-tumor immunity. A high salt diet causes a shift in the host’s immune balance in the pro-inflammatory direction. It induces Th17 cells and M1 type macrophages activation, while interfering with the functioning of Treg cells and M2 type macrophages. This might indicate that a sufficiently high level of sodium in diet during immunotherapy could increase its effectiveness [120].

In rat models, a salt injection leads to the reduction of *Lactobacillus* murinus and to the rise of the Th17 levels. Another research, which was performed on mice models, proved that the high-salt diet leads to a decrease of *Bacteroides* and *Proteobacteria* and to an increase of *Firmicutes* [121].

### 9.5. Glucose Level in the Diet

It is reported that glucose levels have an influence on efficacy of cancer immunotherapy. Glucose is the main source of energy for cancer cells. It has also been shown that the alteration of metabolism in CTLs towards lipid catabolism in a low-glucose tumor environment may increase the efficacy of immunotherapy [111]. However, an important limitation in usage of the low-glucose diet is the fact that the CTLs produce energy in the same way as cancer cells. Because of that, persistent hypoglycemia could impair T lymphocytes’ activity and the production of cytokines by T cells [122]. If this condition lasts for a short period of time, it is reversible. However, if hypoglycemia holds up longer, the inhibition of cytokines production becomes irreversible. Research conducted by Chang et al. indicates that checkpoint inhibitors may be more effective in the treatment of cancers with a high glucose metabolism [123]. There are reports stating that the immune chain blockade might protect lymphocytes T from environmental hypoglycemia [122]. 

Different diets and nutrition models can reduce glucose levels and, thus, weaken tumor proliferative abilities, which can be used during cancer therapy. Calorie restriction (CR) diet is a daily 10–20% reduction in the amount of consumed energy, without causing malnutrition. This diet lowers plasma glucose and insulin levels which, disturbs metabolic pathways. It can also lead to a decrease of insulin-like growth factor-1 (IGF-1). However, it has a therapeutic limitation, since cancer patients often have a problem with cachexia. Intermittent fasting has similar properties. 

Therefore, it seems that the most beneficial treatment for cancer patients, especially those undergoing immunotherapy, is the normalization of glucose levels. Research by Kim et al. on mouse models has shown that the combination of phenformin with PD-1 antibodies increases the effectiveness of this therapy, compared to the use of anti-PD-1 antibodies in monotherapy. In addition, it shows that metformin in combination with anti-PD-1 antibodies does not have such a significant effect on therapy as phenformin. Phenformin exerts a staggering effect on MDSC to suppress the immune response [124].

High glucose levels affect the composition of the microbiome. In people with elevated glucose levels (impaired fasting glycemia, impaired glucose tolerance, diabetes), *A. muciniphila* levels are reduced [125].

In type 2 diabetes, which is most often caused by obesity, the amount of *Faecalibacterium prausnitzii* is decreased. There are also reports stating that the ratio of *Bacteroidetes* to *Firmicutes* correlates with blood glucose levels. In subjects with larger numbers of *Firmicutes*, glucose levels are lower [119].

## 10. Discussion

Through the modulation of the gut microbiota, diet may influence the efficiency of the immunotherapy [20,40,49]. The results will differ based on the subjects’ dietary patterns [40,41,42].

The animal-based diet promotes the growth of *Fusobacterium nucleatum* [41,54,56], which may impair the NK cells’ function, and through that machanism worsen the prognosis in patients with colorectal carcinoma [67]. This speaks for excluding animal products from the cancer patients’ diet. On the other hand, people following an animal-based diet have a lower prevalence of *Roseburia* than people following a plant-based diet [50], which indicates that the animal-based diet could be a better choice after all, due to the *Roseburia’s* negative effect on the immune system [50,54]. We currently lack the data to determine which is more important for the good clinical outcome of the treatmen—a low *Roseburia* level or a low *Fusobacterium nucleatum* level. This issue should be researched further, as it plays an important role in establishing the animal-derived products’ influence on the immunotherapy’s outcome.

Both the animal-based diet [41,42] and the ketogenic [49,57,63] diet are connected to an increased growth of *Bacteroides*. Some of the *Bacteroides* species have a positive influence on the anti-PD-1 treatment’s effectiveness [25,73,76,81,94], whereas others have a negative effect on it [22,54,64,65]. Most sources focus only on the influence of these diets on the whole *Bacteroides* genus without considering the effect they have on the particular species that belong to the *Bacteroides* genus. Future research should focus on establishing which species from that genus are influenced by an animal-based diet and a ketogenic diet, and in what way are they influenced by them. Certain *Bacteroides* species are known to improve patients’ response to the anti-CTLA-4 therapy [34,80,85,89]. This suggests that an animal-based diet or a ketogenic diet could increasse the immunotherapy’s efficiency.

The bacterial species from the *Bifidobacterium* and *Lactobacillus* genera limit the antitumoral response of the cytotoxic T lymphocites [50,54]. However, the two specific *Bifidobacterium* species—*Bifidobacterium breve* and *Bifidobacterium longum*—are known to actually improve the immunotherapy’s results [73,76,81]. This data is relevant for the patients following a plant-based diet, which is known to lower the amounts of *Bifidobacterium* in the intestine [41,43]. We lack the knowledge as to what is more beneficial for the cancer patients treated with immunotherapy—keeping *Bifidobacterium* levels low in order to avoid the suppression of the cytotoxic T lymphocytes or elevating the *Bifidobacterium breve* and *Bifidobacterium longum* amounts due to their supportive properties. This subject should be research further, in order to establish if a plant-based diet could boost or lower the effectiveness of the immunotherapy. Bacteria from the *Lactobacillus* genus are responsible for keeping a tolerogenic state of the mucosa and increase the activity of dendritic cells, both of which support the immunotherapy [19,21,31,32,56,64,66,69,72,92]. Various *Lactobacillus* species are found in certain food products and serve as probiotics [18,45,46,47]. Including these food products into the patient’s diet could be an easy way to improve the immunotherapy’s outcome, under the condition that the positive effects caused by *Lactobacillus* are not canceled out by its ability to limit the cytotoxic T-cells’ activity. 

A diet low in carbohydrates limits the *Roseburia* [49,54] growth, and, because of that, it could support the immunotherapy. On the other hand, a diet rich in complex carbohydrates increases the amounts of *Bifidobacteria* and *Lactobacillus* in the gut [49,54,57], which—as explained in the paragraph above—, under the right circumstances, could strongly support the effectiveness of the immunotherapy. Based on the current knowledge, it is hard to determine which one of these dietary patterns is optimal for the cancer patients treated with immunotherapy. It is also important to remember that there are many versions of both the low-carb and the high-carb diet. All of them should be researched in detail, in order to establish the optimal carbohydrates intake, as well as to find the best source of carbohydrates, for the cancer patients. 

The next important dietary factor that influences the effectiveness of the immunotherapy is the calorie intake. A prolonged excessive calorie intake leads to obesity [108,109,118] and lower *Bacteroidetes* levels [119]. The latter has been linked to a higher risk of colitis caused by CTLA-4 therapy [21]. Weight loss leads to higher *Bacteroidetes* levels [119]. Since a calorie restriction can also increase the effectiveness of the immunotherapy on its own [111,122,124], it could be a good treatment-supporting method for the overweight cancer patients. On the other hand, one must not forget that a prolonged calorie restriction could impair the T lymphocyte’s activity and block the cytokines’ production. This subject should be researched further in order to define a calorie restriction that could be beneficial for patients and an optimal duration of said calorie restriction. 

The Mediterranean diet must be researched further, in order to determine its influence on the *Firmicutes* levels [54,60], which could affect the immunotherapy’s effectiveness, due to its connection with a higher risk of colitis caused by CTLA-4 therapy [21]. 

Other diet-related factors that influence the immune system are vitamins. Vitamin D deficiency and vitamin B1 deficiency impair the immune system [105,110]. Furthermore, vitamin D positively affects the nivolumab’s concentration [100]. It is important to control the vitamin D and vitamin B1 levels in cancer patients receiving immunotherapy. The vitamin D levels’ control is especially important in patients following a vegetarian, vegan, or a fruit-based diet, all of which increase the risk of developing a vitamin D deficiency [56].

The salt intake is also considered to be a diet-related factor that can affect the efficiency of the immunotherapy [120]. This topic should be investigated further in order to gather more data on the subject, since the knowledge we currently have on it is rather limited.

Lastly, we must not forget that the gut microbiota may respond differently to a specific diet, depending on the conditions the patient is suffering from [49,57,63]. Other illnesses must be considered when gathering and interpreting the data.

It is important to point out that the treatment methods discussed in this review do not always give satisfying results, and, when a response occurs, it may not be permanent and it might be heterogeneous [21,30]. It could be correlated with patients’ lifestyle—their diet, sleep cycle, exercise—and used medication. 

One must remember that the differences in cancer biology, cancer staging, and cancer grading are the main factors responsible for the varied treatment response [69,88,89]. 

## 11. Conclusions

The patients’ diet can be an important factor influencing their response to the immunotherapy [20,40,49]. Although the effects that some dietary factors (e.g., vitamin D) have on the immunotherapy’s effectiveness are fairly well known [100,105], there are still many dietary factors (e.g., carbohydrates intake) whose influence on the immunotherapy’s results must be extensively researched. The information we currently have is not enough to fully determine how the different diets affect the immunotherapy’s effectiveness. We propose to gather as much information as possible about the cancer patients’ previous and current eating habits, as well as the reached treatment results, in a search for a diet-related treatment response pattern. We also recommend a more detailed research on how different diets affect certain bacterial species in the gut microbiome in order to better determine their influence on the immunotherapy’s effectieveness. Lastly, for diets that promote the growth of both the immunotherapy-counteracting bacteria and the immunotherapy-supporting bacteria, we suggest a detailed evaluation of all affected bacteria and their meaning to the immunotherapy process, in order to specify if said diets support or counteract the immunotherapy.

## Figures and Tables

**Table 1:** The influence of animal- and plant-based diet on the composition of gut microbiota [41,42,43,50,54,56,57].

Type of Diet	Bacteria Predominant in the Gut Microbiome	Bacteria with Reduced Numbers in the Gut Microbiome
Animal-based diet	*Bacteroides*, *Clostridia*, *Bilophila wadsworthia*, *Fusobacterium nucleatum*.	*Roseburia*, *Eubacterium Rectale* In overweight patients:
		*Roseburia*
		*Collinsella aerofaciens* *Enteroccocus rectale*.
Plant-based diet	*Firmicutes*, *Proteobacteria*, *Ruminococcus* *Roseburia*,	*Enterobacteriaceae*, *Bacteroides*, *Bifidobacterium*.
	*Lb. plantarum*, *Haemophilus*, *Neisseria*, *Aggregatibacter*, *Veionella*.	

**Table 2:** The influence of carbohydrates and their replacements on the composition of gut microbiota [48,49,54,57].

Type of Diet/Product	Bacteria Predominant in the Gut Microbiome	Bacteria with Reduced Numbers in the Gut Microbiome
Low-carb diet	*---*	*Roseburia*, *Eubacterium Rectale* In overweight patients:
		*Roseburia*
		*Collinsella aerofaciens* *Enteroccocus rectale*.
Diet rich in complex carbohydrates	*Bifidobacterium*,	*Enterobacteriacae*.
	*Lactobacillus*.	
Diet rich in sugar	*Clostridium difficile*, *Clostridium prefringens*.	*---*
	Exessive sugar intake might also lead to *Candida* overgrowth.	
Artificial sweeteners	*Bacteroides*	*Lactobacillus reuteri*
